# Tooth avulsion accidents due to urgent and emergency orotracheal intubation

**DOI:** 10.4317/medoral.23375

**Published:** 2020-02-10

**Authors:** Anna Karyna F de Carvalho Galvão, Gloria Maria Pimenta Cabral, Alexandre Franco Miranda, Fernando Martins Baeder, Maria Teresa Botti Rodrigues Santos

**Affiliations:** 1 DDS, PhD. Graduate Professor, Dentistry for Special Needs Individuals and Hospital Dentistry, Higher Education Institute of Paraíba (IESP), João Pessoa, PB, Brazil; 2 MSc, PhD. Graduate Professor, Dentistry for Special Needs Individuals and Hospital Dentistry, Higher Education Institute of Paraíba (IESP), João Pessoa, PB, Brazil; 3 DDS, MSc, PhD. Postgraduate Program Stricto Sensu in Gerontology, Department of Geriatric Dentistry, Dentistry for Special Patients and Hospital Dentistry - Catholic University of Brasília (UCB), Brazil; 4 DDS, PhD. Graduate Professor, Cruzeiro do Sul University São Paulo, SP, Brazil; 5 DDS, MSc, PhD. Associate Professor, Special Needs Individuals, Postgraduate Program in Dentistry, Cruzeiro do Sul University São Paulo, SP, Brazil

## Abstract

**Background:**

Intubation is necessary during critical situations to reduce the risk of death. In Brazil, a need exists to determine the prevalence of tooth avulsions in emergency and urgent care. The objective of this study was to identify the causes of orotracheal intubation (OTI), the number of tooth avulsions, and the avulsed teeth that result from urgent and emergency intubation.

**Material and Methods:**

The sample consisted of 116 patients (total group) in intensive care units (ICUs) distributed across Group 1 (G1), which was composed of 71 patients from an urgent-care hospital, and Group 2 (G2), which was composed of 45 patients from an emergency hospital. Clinical examinations showed dental alveolus with signs of recent exodontia in the upper and lower anterior regions. Sociodemographic data and the reason for intubation were evaluated. The Shapiro-Wilk normality test, chi-square test, Fisher’s exact test, Mann-Whitney U test, and univariate logistic regression were performed with a significance level of 5%.

**Results:**

The avulsion prevalence was 4.3%, with more cases receiving emergency intubation (n=4). All avulsions occurred in adults, and a significant difference (*p*=0.011) was observed with regard to the elderly. A 1-year reduction in age increased the chance of tooth avulsion during intubation by 1.09 times; being female increased the chance by 2.88 times.

**Conclusions:**

Pulmonary problems were the major causes of intubation, with the highest tooth avulsion prevalence observed during emergency intubation. The avulsed teeth were 11, 12, 13, 22, 32, and 33 across all cases.

** Key words:**Tooth avulsion, tooth injuries, intubation, intensive care units, emergencies.

## Introduction

Dental trauma is a complication that can occur during orotracheal intubation (OTI) via laryngoscopy (1-5) or during anesthetic maintenance (i.e., sedation) (6), extubation (5,7), or some combination therein. The most frequent medicolegal complaints related to intubation are injuries to the teeth, attributing to the most complaints against medical professionals for poor medical practice (1) however, dental injuries can occur under the care of experienced and careful professionals (1). The avulsion of permanents teeth is the most serious dental injury, and rapid and correct intervention is fundamental for favorable prognoses in the resolution of these cases (8-9).

A difficult airway accesses is a trauma risk predictor to tooth injury during intubation procedure (4,10-11). The teeth and supporting structural conditions are also important because crowned teeth, extensive restorations, and periodontal diseases predispose the patient to damage during intubation (12). Thus, a preoperative evaluation should be performed whenever possible to identify these trauma risk predictors (6,12-13).

The most affected teeth are the upper and lower anterior teeth, especially the upper central incisors (3,14). Some dental trauma preventive measures have been adopted such as the use of mouth guards during laryngoscopy (1,3,5,12-13,15) or the placement of an adhesive on the laryngoscope blade (7) that acts as a shock absorber by uniformly distributing the forces produced when using the laryngoscope (5).

Oral damage during OTI is not completely prevenTable and should be assumed by the physician and the patient as an inherent risk of the clinical procedure (1,10,12). In the event of trauma, many professionals are indicted for negligence, and proving that the physician provided the elementary healthcare needed can be difficult and expensive (1). Close cooperation between dental surgeons and physicians has been advocated, although it does not eliminate the risk of trauma (1,3,14).

After conducting a literature search of the articles published up to the present date combining the terms tooth avulsion, tooth injuries, and OTI, it were found 18 studies, however none of them evaluated the patient in a hospital environment after OTI in an intensive care unit (ICU) in Brazil. Therefore, the aim of this study was to determine the causes of OTI, identify patient comorbidities, the number of tooth avulsions, and the avulsed teeth resulting from urgent and emergency OTI. The study hypothesized that more tooth avulsion accidents would occur during emergency OTI.

## Material and Methods

This cross-sectional study was conducted from July to December 2018 on patients admitted to the ICU of Senator Humberto Lucena Trauma Hospital (Hospital do Trauma Senador Humberto Lucena) and Lauro Urquiza Wanderley State Emergency Hospital (Hospital Estadual de Emergência Lauro Urquiza Wanderley), both of which are located in João Pessoa, Paraiba, Brazil.

Group 1 (G1) consisted of patients from the private hospital, which is accredited with Level 3 Excellence by the National Accreditation Organization (Organização Nacional de Acreditação; ONA). This hospital provides clinical and urgent-care (medical care provided for illnesses or injuries which require prompt attention) among others. It has dental surgeons working from Monday through Saturday from 7:00 a.m. to 1:00 p.m. Group 2 (G2) was composed of patients from the state public hospital, which has a Level 1 Accreditation certificate by the ONA and is a reference center for urgent and emergency (an unforeseen combination of circumstances or the resulting state that calls for immediate action) trauma care. This hospital serves the population of the greater João Pessoa area and other regions of the state. A dental surgeon is a part of the clinical multidisciplinary team that is present each day for 24 hours.

To collect the data, two weekly visits were performed on different days over a 3-month period for both hospitals, with 24 visits total for each hospital. Only one dental surgeon (AKFCG) assessed all patients.

A total of 180 individuals from both hospitals took part in this study. The inclusion criteria were as follows: patients in the ICU who underwent urgent (G1) or emergency (G2) OTI and had their anterior teeth. Patients were excluded if they had suffered trauma to the oral cavity region prior to intubation.

Of the patients who met the eligibility criteria, 64 were excluded. A total of 46 were excluded from G1 (21 did not have their anterior teeth, and 25 did not require intubation), and 18 were excluded from G2 (12 did not have their anterior teeth, four did not need intubation, and two suffered from trauma in the buccal region).

The final sample of G1 included 71 patients from the general ICU (10 beds) or the clinic (7 beds). G2 consisted of 45 patients from the adult ICU (16 beds).

Initially, all patients from the ICU were evaluated, and those with orotracheal tubes were selected. To determine the occurrences of tooth avulsion, a clinical examination with direct visualization of the oral cavity was performed, with respect to the inclusion/exclusion criteria.

The presence of dental alveolus with signs of recent exodontia (clot in the bony socket) in the region of the upper [11-13,21-23] or lower [31-33,41-43] anterior teeth was verified. After this evaluation, the sociodemographic profile (gender and age), type of hospital where the data were collected (private, public, urgent, or emergency), OTI, and the presence of associated comorbidities were collected from patients’ medical records.

For the analyses, the data were tabulated and analyzed using descriptive and inferential statistics. The Shapiro-Wilk normality test, Chi-square test, Fisher's exact test, Mann-Whitney U test, and Univariate logistic regression with a 5% significance level were performed using IBM SPSS version 21.0.

## Results

A total of 116 individuals receiving OTI participated in this study. Of these patients, the majority was male (n=75, 64.7%), 71 (61.2%) patients were treated at an urgent-care unit, and 45 (38.8%) were treated at an emergency unit. The mean age of the patients treated at the urgent-care unit was 68.8 (±15.9) years, the majority of which was male (n=39, 54.9%). The mean age of the patients treated at the emergency unit was 47.9 (±20.2) years, the majority of which was also male (n=36, 80.0%). Table 1 shows the distribution of the patients by age group and gender. The majority of G1 was elderly, whereas G2 was composed of younger adults. Both G1 and G2 were primarily male (Table 1).

**Table 1:**
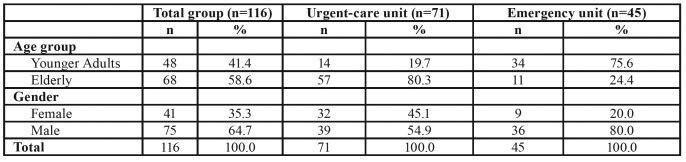
Distribution of patients by age group and gender.

The major reason for the intubation of the 116 patients was pulmonary disease related, both for the patients treated in the urgent-care and emergency units. The major comorbidity presented by all of the participants were the chronic systemic diseases. The complete distribution of the reasons for intubation among the 116 patients and those treated at the urgent-care and emergency units as well as their associated comorbidities are presented in Table 2.

Tooth avulsion was observed in five (4.3%) patients, with one (20.0%) woman at the urgent-care unit with the avulsion of teeth 11 and 32, and four cases (80.0%; two women and two men) in the emergency unit with the avulsion of teeth 12, 13, 22, and 33.

Regarding the total number of patients evaluated, an association was observed between age and the occurrence of tooth avulsion (*p*=0.005); furthermore, younger patients were more likely to be treated at the emergency unit (*p*=0.010). The mean ages ​​of the different groups evaluated are shown in Table 3.

**Table 2:**
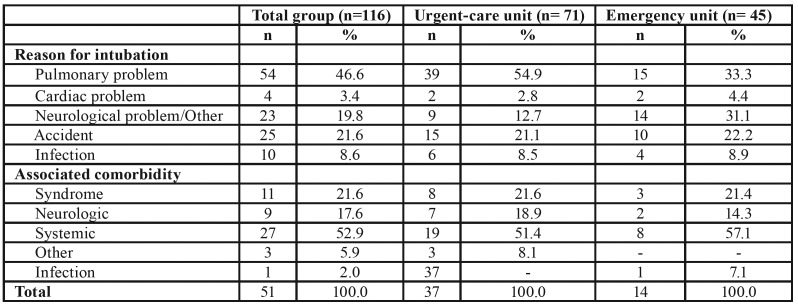
Distribution of the reasons for intubation and associated comorbidities among the evaluated patients.

**Table 3:**
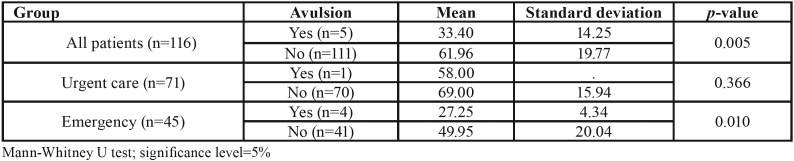
Mean ​​(±SD) age of patients with avulsed teeth.

**Table 4:**
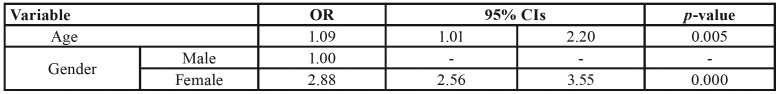
Linear regression of the occurrence of tooth avulsion by age and gender.

All avulsions occurred in younger adult patients, with a significant difference between these adults and the elderly (*p*=0.011). Every 1-year reduction in age increased the patient’s chance of having tooth avulsion during OTI by 1.09 times (95% CIs=1.01 - 2.20). Women were 2.88 times more likely to have tooth avulsion than men (95% CIs=2.56 - 3.55; Table 4).

Of the 51 individuals who presented with a comorbidity, the only patient who suffered from tooth avulsion presented with diabetes mellitus as a systemic alteration (avulsed teeth 11 and 32). The other three trauma patients had no associated comorbidities.

## Discussion

This study is the first to clinically evaluate the presence of tooth avulsion accidents due to OTI during urgent and emergency care with regard to hospitals in João Pessoa, PB, Brazil. Traumatic injuries to the upper airways are common during OTI and can involve the dental organs (3,10,14).

Few studies have reported the occurrence of dental trauma during OTI. Two of these studies evaluated the medical records completed by anesthesiologists with retrospective analyses (1,10). Others evaluated dental and airway pre-intubation conditions (14,16), and another compared the types of laryngoscopes used for intubation (17). Four case reports described accidents during OTI. Feltracco *et al*. (18) described the displacement of dental bridge to pharyngeal recess. Ozer *et al*. (19) described the avulsion of the four upper incisors that moved to the nasopharynx area during OTI performed with a Macintosh laryngoscope. Feltracco *et al*. (20) reported two cases of dental injuries. In the first, the patient blamed prosthesis movement after OTI, and the second reported that the crown of tooth 11 had fractured. In both cases, the patients required compensation for the damages caused. Tammara *et al*. (21) described an emergency OTI in which a tooth was found in the trachea after a chest X-ray. In all of the aforementioned studies, physicians performed the oral cavity evaluations. Darawade *et al*. (5) proposed that a dentist should perform the clinical examination of the oral cavity before and after OTI because he or she is the professional most qualified to conduct a thorough evaluation.

The possibility of dental trauma occurring during intubation (the silent trauma) is well known. Emergencies happen and even experienced partitions can be placed in awkward situations. Modern methods of intubation result in in negligible dental risk (0.13%) (10). A relatively high proportion of avulsions was observed in this study (4.3%) what may reflect specific practices realized in the hospitals evaluated.

No studies have compared the prevalence of tooth avulsion with regard to urgent and emergency OTI. However, some studies have compared the injuries caused during the intubation process of elective and emergency surgeries (1,10,18). Emergency OTI is more likely to cause injury than elective OTI (18). However, Gaudio *et al*. (1) conducted two retrospective studies and observed that 10% of the injuries occurred during emergency surgeries and that 90% occurred during elective surgeries. Furthermore, Gaudio *et al*. (10) verified that injuries occurred in 75% of elective surgeries and in 10% of emergency surgeries. However, the authors did not justify these results in either study.

Regarding traumatic injuries, Adolphs *et al*. (12) observed a prevalence of 0.02%; however, these authors did not describe the type of injury. Martin *et al*. (11) reported a prevalence of 0.2% but did not describe the type of trauma nor the teeth affected. Mourão *et al*. (13) reported a 25% tooth trauma rate among patients receiving OTI but did not distinguish the type of injury. In this study, the prevalence of tooth avulsion trauma caused by OTI was 4.3%. This prevalence was higher than the results reported by Gaudio *et al*. (10) (0.13%), Adolphs *et al*. (12) (0.02%), Martin *et al*. (11) (0.2%), and Kuo *et al*. (22) (0.108%) and was lower than the result reported by Mourão *et al*. (13) (25%). Importantly, retrospective studies of medical records (1,10) did not describe the exclusion of incomplete medical records. Dental treatment prior to OTI was indicated when dental alterations that predisposed to previous trauma were detected (22-23). Furthermore, these evaluations were not performed by a professional dental surgeon responsible for this area of ​​knowledge.

The avulsed teeth observed in this study were 11, 12, 13, 22, 32, and 33, which corroborates with the extant literature (7,10,16,21,22). However, other teeth can suffer trauma, including 41, 22, 31, 41, 42, and 43 (14).

A difference was observed between the studied groups with regard to age: G1 was mostly composed of elderly patients, whereas G2 was composed of younger adults. We infer from these results that the socioeconomic level of the evaluated patients played a role. G1 patients had private health insurance and rapid access to treatment, which reduced the likelihood of progressing to a more severe condition, except among the elderly who had associated comorbidities. G2 patients were treated at a public hospital; because of the difficulty of obtaining examinations and consultations at the secondary healthcare level, these patients (especially those facing the Brazilian public health situation) are at risk for aggravated health conditions.

According to Gaudio *et al*. (10) almost two-thirds of tooth injuries during OTI occur in patients aged 45-65 years because these patients require surgical procedures more often than the young. Azeredo *et al*. (14) showed that with increasing age, patients are more prone to upper airway injuries during the OTI process. Patients aged 80 years old and over are more subject to injury, including dental injury, than those aged 40-49 years. However, Kuo *et al*. (22) reported that 85% of the dental injuries occur in patients aged 40-49 years old. In the present study, the patients in G2 needed to undergo emergency OTI despite being adults (which presupposes less physiological bone loss, fewer periodontal problems, and consequently better dental conditions). This event increased the chance of tooth avulsion in both genders, corroborating the results described in the literature (1).

Regarding the use of preventive measures for urgent or emergency OTI, a blocked airway and teeth that are susceptible to trauma are difficult to predict at the time of the procedure because the patient requires rapid intervention given that as his or her life is at risk. The Macintosh laryngoscope is the most commonly used such device worldwide, and it is associated with injuries that occur during laryngoscopy; however, other factors should be considered (1,13,17). Mourão *et al*. (2) found that 38.6% of a group of patients subjected to the Macintosh laryngoscope experienced dental injuries. In cases of a blocked airway, a safer option is to use a video laryngoscope because it causes less trauma (1,17,21), as it does not require greater jaw traction for the visualization of the glottis; however, it has a high cost. Another method of preventing dental trauma is the use of mouth guards (prefabricated or individually made with addition silicone) at the time of intubation (3,10,12-13,15,22). In some cases (e.g., mouth opening limitations and severe tooth mobility), however, this use is contraindicated (10). When the tooth is isolated, the use of a suture thread for anchoring the nearest teeth is indicated (16,22). Although the use of oral devices is a form of tooth protection, patients who undergo emergency OTI do not benefit from the use of this device (1). However, additional studies on this subject are required.

The limitation of this study was not having evaluated the patient prior to OTI; thus, we could not verify the occurrence of other tooth injuries.

The treatment of a permanent tooth avulsion is its immediate replantation because the later it is performed, the worse the prognosis (3,9,10,24-25). However, would any case of avulsion in an ICU be indicated for dental replantation? Would a patient on corticosteroids, high dose anticoagulants, or bisphosphonate with uncontrolled diabetes mellitus, who was hemodynamically unsTable, had sepsis, or nosocomial infection be indicated for invasive treatment? Replantation in these cases should be evaluated as should all systemic conditions, associated diseases, comorbidities, medications used, and hematological exams to verify the best approach for each case. In Brazil, the presence of a dentist has been compulsory since 2013 (26) when the Ministry of Health recognized the relevance of a qualified dentistry professional with clinical experience as a member of the multidisciplinary team in the ICU. In some cases, dental replantation might be indicated; in others, however, this option will not be the best treatment. It is the responsibility of the dentist to evaluate the patient’s overall clinical condition, medications in use, and laboratory tests to decide the best approach for each case.

## Conclusions

Chronic pulmonary insufficiency was the most prevalent cause of OTI, with a greater number of avulsion accidents occurring in adults during an emergency procedure (teeth 12, 13, 22, and 33) compared with those during an urgent-care procedure (teeth 11 and 32).

Pulmonary problems were the major causes of intubation, with the highest tooth avulsion prevalence observed during emergency intubation. The avulsed teeth were 11, 12, 13, 22, 32, and 33 across all cases.
